# Supplementary material for: Insights into PCSK9-LDLR Regulation and Trafficking via the Differential Functions of MHC-I Proteins HFE and HLA-C
Source: Cells. 2024 May 17;13(10):857. doi: 10.3390/cells13100857 (PMC11119474; doi:10.3390/cells13100857)
Supplement: Supplementary file 1 [file cells-13-00857-s001.zip › cells-2940739-supplementary.pdf]

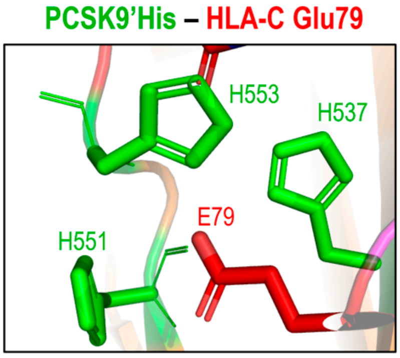

Figure S1. Graphic representation of the 3 Histidine residues in the PCSK9′ CHRD (green) in contact with Glu79 of HLA-C (red).

A

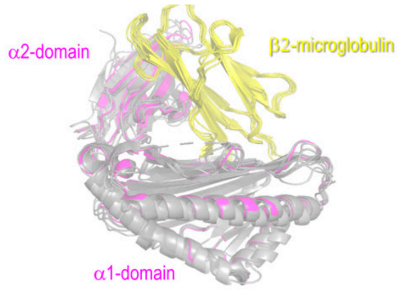

Q30201 · HFE_HUMAN PDB : 1A6Z
P10321 · HLA-C_HUMAN PDB : 6JTO
Q53Z42 · HLA-A2_HUMAN PBD : 6TRN
P01889 · HLA-B_HUMAN PDB : 1M05
P30511 · HLA-F_HUMAN PDB : 5IUE
P17693 · HLA-G_HUMAN PDB : 3KYO

B

```
HFE. _10001  1    --------MGPRARPALLLLMLLQTAVLQ-----------------GRLLR-------------SHSLHYLFMGASEQD  41
HLA-C_10002  1    -----MRVMAPRALLLLLSGGLALTETWA----------------------------------CSHSMRYFDTAVSRPG  40
HLA-A_10003  1    -----MAVMAPRTLLLLLSGALALTQTWA----------------------------------GSHSMRYFFTSVSRPG  40
HLA-B_10004  1    -----MLVMAPRTVLLLLSAALALTETWA----------------------------------GSHSMRYFYTSVSRPG  40
HLA-E_10005  1    -MEPS-FYSSRRPWPLPRPGRVSAGSGWKRPLPGVERGRPGGGEGLGEPRREEGRADLSPSSPPGSHSLKYFHTSVSRPG  78
HLA-F_10006  1    --------MAPRSLLLLLSGALALTDTWA----------------------------------GSHSLRYFSTAVSRPG  37
HLA-G_10007  1    MKTPRMVVMAPRTLFLLLSGALTLTETWA----------------------------------GSHSMRYFSAAVSRPG  45

                                                                α1 domain
HFE. _10001  42   LGLSLFEALGYVDDQLFVFYDH--ESRRVEPRTPWVSSRISSQMWLQLSQSLKGWDHMFTVDFWTIMENHNHSK-ESHTL  118
HLA-C_10002  41   RGEPRFISVGYVDDTQFVRFDSDAASPRGEPRAPWVEQEG-PEYWDRETQKYKRQAQADRVSLRNLRGYYNQSEDGSHTL  119
HLA-A_10003  41   RGEPRFIAVGYVDDTQFVRFDSDAASQRMEPRAPWIEQEG-PEYWDQETRNMKAHSQTDRANLGTLRGYYNQSEDGSHTI  119
HLA-B_10004  41   RGEPRFISVGYVDDTQFVRFDSDAASPREEPRAPWIEQEG-PEYWDRNTQIYKAQAQTDRESLRNLRGYYNQSEAGSHTL  119
HLA-E_10005  79   RGEPRFISVGYVDDTQFVRFDNDAASPRMVPRAPWMEQEG-SEYWDRETRSARDTAQIFRVNLRTLRGYYNQSEAGSHTL  157
HLA-F_10006  38   RGEPRYIAVEYVDDTQFLRFDSDAAIPRMEPREPWVEQEG-PQYWEWTTGYAKANAQTDRVALRNLLRRYNQSEAGSHTL  116
HLA-G_10007  46   RGEPRFIAMGYVDDTQFVRFDSDSACPRMEPRAPWVEQEG-PEYWEEETRNTKAHAQTDRMNLQTLRGYYNQSEASSHTL  124

                                                                  α2 domain
HFE. _10001  119  QVILGCEMQEDN-STEGYWKYGYDGQDHLEFCPDTLDWRAAEPRAWPTKLEWERHKIRARQNRAYLERDCPAQLQQLLEL  197
HLA-C_10002  120  QRMSGCDLGPDGRLLRGYDQSAYDGKDYIALNEDLRSWTAADTAAQITQRKLEAARAAEQ-LRAYLEGTCVEWLRRYLEN  198
HLA-A_10003  120  QIMYGCDVGPDGRFLRGYRQDAYDGKDYIALNEDLRSWTAADMAAQITKRKWEAVHAAEQ-RRVYLEGRCVDGLRRYLEN  198
HLA-B_10004  120  QSMYGCDVGPDGRLLRGHDQYAYDGKDYIALNEDLRSWTAADTAAQITQRKWEAAREAEQ-RRAYLEGECVEWLRRYLEN  198
HLA-E_10005  158  QWMHGCELGPDGRFLRGYEQFAYDGKDYLTLNEDLRSWTAVDTAAQISEQKSNDASEAEH-QRAYLEDTCVEWLHKYLEK  236
HLA-F_10006  117  QGMNGCDMGPDGRLLRGYHQHAYDGKDYISLNEDLRSWTAADTVAQITQRFYEAEEYAEE-FRTYLEGECLELLRRYLEN  195
HLA-G_10007  125  QWMIGCDLGSDGRLLRGYEQYAYDGKDYLALNEDLRSWTAADTAAQISKRKCEAANVAEQ-RRAYLEGTCVEWLHRYLEN  203

HFE. _10001  198  GRGVLDQQVPPLVKVTHH-VTSSVTTLRCRALNYYPQNITMKWLKDKQPMDAKEFEPKDVLPNGDGTYQGWITLAVPPGE  276
HLA-C_10002  199  GKETLQRAEPPKTHVTHHPLSDHEATLRCWALGFYPAEITLTWQRDGEDQ-TQDTELVETRPAGDGTFQKWAAVVVPSGQ  277
HLA-A_10003  199  GKETLQRTDPPKTHMTHHPISDHEATLRCWALGFYPAEITLTWQRDGEDQ-TQDTELVETRPAGDGTFQKWAAVVVPSGE  277
HLA-B_10004  199  GKDKLERADPPKTHVTHHPISDHEATLRCWALGFYPAEITLTWQRDGEDQ-TQDTELVETRPAGDRTFQKWAAVVVPSGE  277
HLA-E_10005  237  GKETLLHLEPPKTHVTHHPISDHEATLRCWALGFYPAEITLTWQQDGEGH-TQDTELVETRPAGDGTFQKWAAVVVPSGE  315
HLA-F_10006  196  GKETLQRADPPKAHVAHHPISDHEATLRCWALGFYPAEITLTWQRDGEEQ-TQDTELVETRPAGDGTFQKWAAVVVPPGE  274
HLA-G_10007  204  GKEMLQRADPPKTHVTHHPVFDYEATLRCWALGFYPAEIILTWQRDGEDQ-TQDVELVETRPAGDGTFQKWAAVVVPSGE  282

HFE. _10001  277  EQRYTCQVEHPGLDQPLIVIWEPSPSGTL-VIGVISGIAVFVVI-LFIGILFIILRKRQGSRGAMGHYVLAERE------  348
HLA-C_10002  278  EQRYTCHMQHEGLQEPLTLSWEPSSQPTIPIMGIVAGLAVLVVLAVLGAVVTAMMCRRKSSGGKGGSCSQAACSNSAQGS  357
HLA-A_10003  278  EQRYTCHVQHEGLPKPLTLRWELSSQPTIPIVGIIAGLVLLGAV-ITGAVVAAVMWRRKSSDRKGGSYTQAASSDSAQGS  356
HLA-B_10004  278  EQRYTCHVQHEGLPKPLTLRWEPSSQSTVPIVGIVAGLAVLAVV-VIGAVVAAVMCRRKSSGGKGGSYSQAACSDSAQGS  356
HLA-E_10005  316  EQRYTCHVQHEGLPEPVTLRWKPASQPTIPIVGIIAGLVLLGSV-VSGAVVAAVIWRKKSSGGKGGSYSKAEWSDSAQGS  394
HLA-F_10006  275  EQRYTCHVQHEGLPQPLILRWEQSPQPTIPIVGIVAGLVVLGAV-VTGAVVAAVMWRKKSSDRNRGSYSQAAAYSVVSGN  353
HLA-G_10007  283  EQRYTCHVQHEGLPEPLMLRWKQSSLPTIPIMGIVAGLVVLAAV-VTGAAVAAVLWRKKSSD-----------------  343

HFE. _10001       --------------------------------------------------------------------------------
HLA-C_10002  358  DESLITCKA-----------------------------------------------------------------------  366
HLA-A_10003  357  DVSLTACKV-----------------------------------------------------------------------  365
HLA-B10004  357  DVSLTA--------------------------------------------------------------------------  362
HLA-E_10005  395  ESHSL---------------------------------------------------------------------------  399
HLA-F_10006  354  LMITWWSSLFLLGVLFQGYLGCLRSHSVLGRRKVGDMWILFFLWLWTSFNTAFLALQSLRFGFGFRRGRSFLLRSWHHLM  433
HLA-G_10007       --------------------------------------------------------------------------------

HFE. _10001       ---------
HLA-C_10002       ---------
HLA-A_10003       ---------
HLA-B_10004       ---------
HLA-E_10005       ---------
HLA-F_10006  434  KRVQIKIFD  442
HLA-G_10007       ---------
```

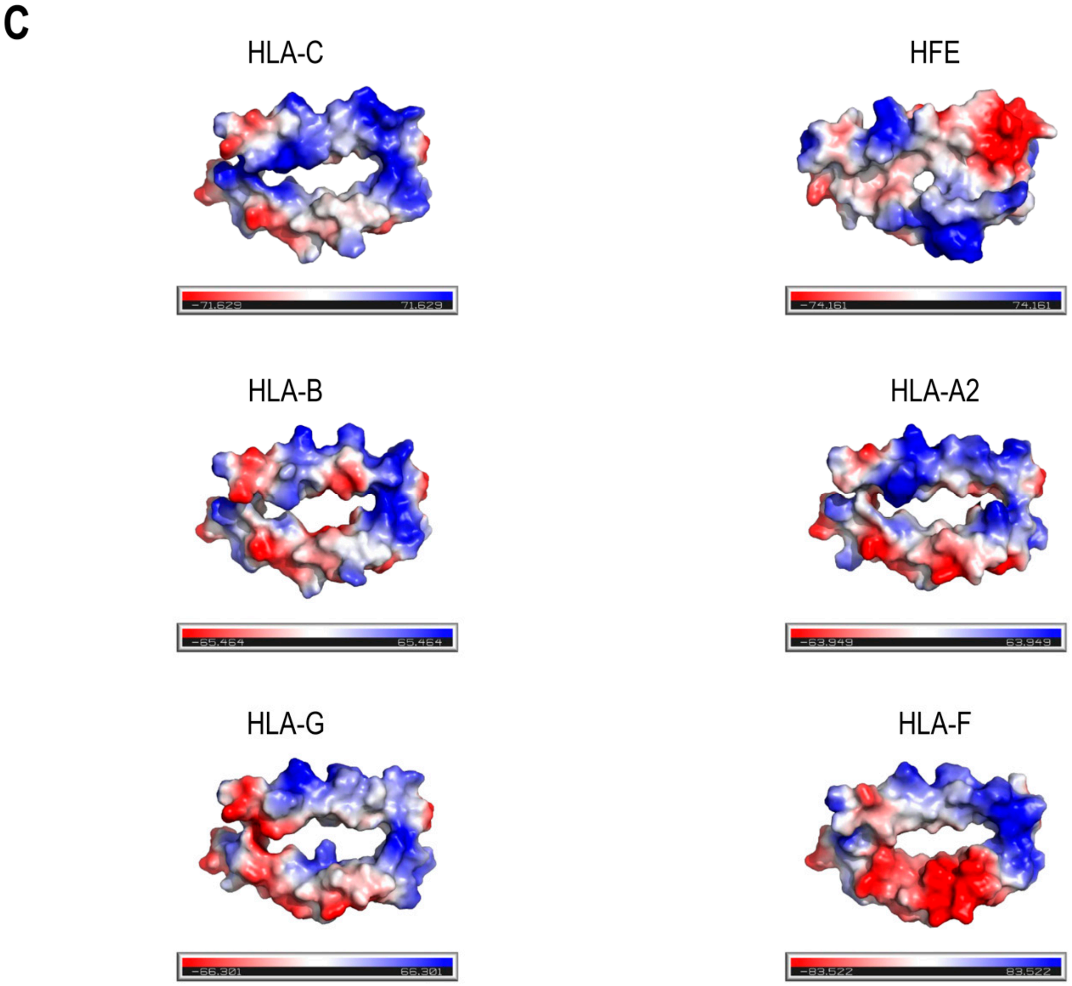

Figure S2. Structure and interaction sites of all MHC-I molecules. (A) Superimpose of the structures of the different MHC-I molecules: HFE PBD:1a6z; HLA-C PDB: 6jto (pink); HLA-A2 PBD: 6trn; HLA-B PDB: 1m05; HLA-F PDB: 5iue; HLA-G PDB: 3kyo. (B) Sequence alignment of MHC-I molecules and visualization of basic residues (highlighted in blue) and acidic residues (highlighted in red) of the antigenic pocket of MHC-I molecules. (C) Electrostatic potential of the antigenic pocket of MHC-I molecules.

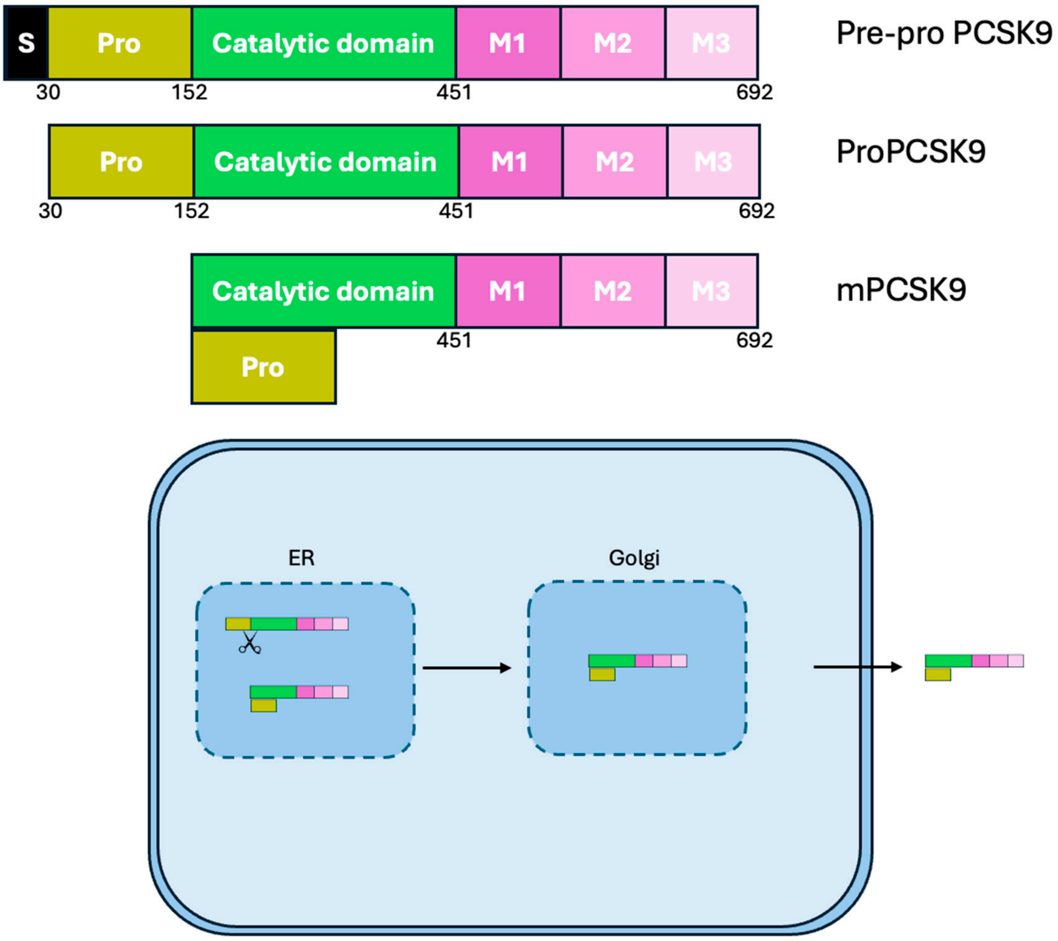

Figure S3. The schematic of different domains of PCSK9 and its trafficking in cells. The processing of PCSK9 starts with the synthesis of the 72-kDa preproPCSK9 protein (comprising 692 aa) in the ER, following with the cleavage of the signal peptide. Upon cleavage of signal peptide, proPCSK9 undergoes autocatalytic cleavage at its N-terminal (at VFAQ152↓ motif) in the ER. Nevertheless, the cleaved prodomain permanently stays non-covalently attached to the rest of the protein, even when it is secreted into the extracellular media. The heterodimer complex of PCSK9 includes a 14 kDa propeptide (aa 31-152) and a glycosylated 63 kDa (aa 153-692) mature protein (mPCSK9) that is transported to the Golgi and then secreted to the outer media.
